# Grade IV frostbite requiring bilateral below knee amputations: a case report

**DOI:** 10.1186/1757-1626-2-6635

**Published:** 2009-04-08

**Authors:** Michael J Ramdass

**Affiliations:** 1 Department of Vascular Surgery, Medway Maritime Hospital, Windmill Road, Gillingham, Kent ME7 5NY, England

## Abstract

A rare case of grade IV frostbite is presented resulting in bilateral below knee amputations. This case highlights the importance of early versus late amputation as well as the importance of close collaboration between the rehabilitation, surgical, psychosocial, and public health disciplines in this rare and challenging problem that still may be encountered in the United Kingdom.

## Case presentation

**Figure 1:**
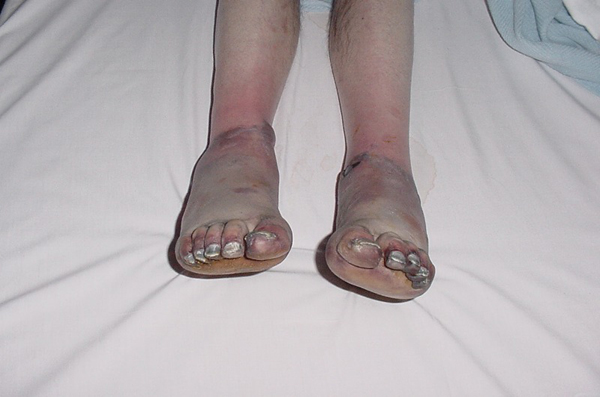
**Initial presentation of frostbite on day 2**.

**Figure 2:**
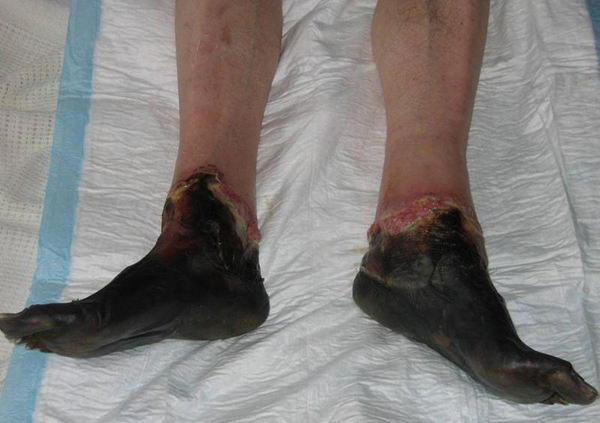
**Appearance of grade IV frostbite with completely mummified feet 3 weeks post-injury**.

This is the case of a 50-year old homeless man who sustained grade IV frostbite injuries to both feet after sleeping out in sub-zero temperatures. The patient first presented with ischaemic feet with well demarcated areas around the ankle (Figure 1). The feet were left to demarcate and became mummified (Figure 2), then bilateral below knee amputations were performed.

Delayed amputation had a positive effect on psychological acceptance of bilateral amputations by the patient as well as preparation for rehabilitation.

Bilateral below-knee amputations are rarely performed. The predisposing factors include diabetic foot sepsis, atherosclerosis, frostbite, burns, trauma, calciphylaxis-related gangrene, suicide attempt, sensory loss and calf-wound healing failure after coronary revascularisation [1,2]. One of the main issues surrounding this problem is the justification of restorative efforts due to a high failure rate.

Frostbite can be graded into stages I to IV depending on tissue depth involvement. Two main approaches can be adopted with deep frostbite, either (a) early necrectomy with subsequent local treatment of wounds and (b) long-term conservative treatment until formation of demarcation line of necrotic tissue with subsequent resection and formation of a stump to facilitate rehabilitation.

This case highlights the importance of careful consideration of early versus late amputation as well as the importance of close collaboration between the rehabilitation, surgical, psychosocial, and public health disciplines in this rare and challenging problem that still may be encountered in the United Kingdom.

## Consent

Written informed consent was obtained from the patient for publication of this case report and accompanying images. A copy of the written consent is available for review by the Editor-in-Chief of this journal.

## Competing interests

The author declares that he has no competing interests.

## Author's Contribution

MJR is the sole author to this article and has written and researched the case himself.
